# Effect of SARS CoV2-Neutralizing Monoclonal Antibody on Hospitalization and Mortality in Long-Term Care Facility Residents

**DOI:** 10.14336/AD.2022.0205

**Published:** 2022-10-01

**Authors:** Monika Murillo, Christine Lomiguen, Mark Terrell, Ashley King, James Lin, Silvia Ferretti

**Affiliations:** ^1^LECOM Health - Millcreek Community Hospital, Erie, Pennsylvania, USA.; ^2^LECOM Health - Institute for Successful Aging, Erie, Pennsylvania, USA.; ^3^LECOM Health - Medical Associates of Erie, Erie, Pennsylvania, USA.; ^4^Lake Erie College of Osteopathic Medicine, Erie, Pennsylvania, USA

**Keywords:** COVID-19, SARS-CoV-2, neutralizing monoclonal antibod, hospitalization, mortality

## Abstract

To measure the association between intravenous administration of monoclonal antibody bamlanivimab (LY-CoV555) to long-term care facility (LTCF) residents recently diagnosed with pre-symptomatic, mild-to-moderate COVID-19 and are considered high risk for disease progression with mortality, hospitalization, and adverse effects. A retrospective analysis of LTCF residents with confirmed COVID-19, pre-symptomatic, mild to moderate disease, who were treated with bamlanivimab (LY-CoV555) were compared to similar LTCF residents who did not receive monoclonal antibody treatment. Dependent variables investigated included mortality and hospitalization as primary outcomes with adverse effects as the secondary outcome. A total of 107 residents from three LTCFs were diagnosed with pre-symptomatic, mild-to-moderate COVID-19 between November 1, 2020, and December 31, 2020. Of the 107 study participants, 44 residents provided consent to treatment, of which 39 received a single intravenous infusion of neutralizing monoclonal antibody, bamlanivimab 700mg, early in the disease, and 5 received an incomplete dose. Of the 39 residents who received the full dose of bamlanivimab, 5 (12.8%) were admitted to the hospital and 4 (10.3%) died. Conversely, of the 63 residents who did not receive the monoclonal antibody, 26 (41.3 %) were admitted to the hospital and 18 (28.6%) died. Relative risk for hospitalization and death were statistically significantly lower for those residents who received the full bamlanivimab treatment. No serious adverse effects were documented on any patient. Intravenous administration of monoclonal antibody bamlanivimab (LY-CoV555) to LTCF residents recently diagnosed with pre-symptomatic, mild to moderate COVID-19 was significantly associated with reduced mortality and hospitalization. The monoclonal antibody was well-tolerated.

The coronavirus disease 2019 (COVID-19) pandemic, due to the novel severe acute respiratory syndrome coronavirus 2 (SARS-CoV-2), caused a worldwide, sudden, and substantial increase in hospitalizations for and death from pneumonia with multi-organ disease [1]. Long-term care facility (LTCF) residents were, and continue to be, at particularly high risk for morbidity and mortality associated with infection with SARS-CoV-2, given their age and high prevalence of chronic medical conditions, combined with functional impairment that often requires frequent and close contact with health care providers, who may inadvertently spread the virus to residents (Garg S, 2020; Wortham JM, 2020 COVID-NET). Prior to the advent of COVID-19 vaccinations, healthcare providers applied their best knowledge of treatments to mitigate the spread of disease, hospitalization, and death among vulnerable elderly residents in LTCFs.

Preclinical studies of neutralizing antibody treatments for SARS-CoV-2 infection in several animal models were associated with promising results, including marked reductions in viral loads in the upper and lower respiratory tracts [1]. On November 9, 2020, the United States Food and Drug Administration (FDA) issued an Emergency Use Authorization (EUA) for a single infusion of 700 mg bamlanivimab (LY-CoV555) for the treatment of mild-to-moderate COVID-19 in adults. (FDA authorizes monoclonal antibody for treatment of COVID-19, 2020). Bamlanivimab (LY-CoV555) binds to the receptor binding domain of the spike protein of SARS-CoV-2, blocking the spike protein’s attachment to the human ACE2 receptor. Monoclonal antibodies that target the spike protein have been shown to have a clinical benefit in treating SARS-CoV-2 infection. Preliminary data suggest that monoclonal antibodies may play a role in preventing SARS-CoV-2 infection in household contacts of infected patients and during skilled nursing and assisted living facility outbreaks [5,6]. The purpose of this study was to examine the effect of the virus-neutralizing monoclonal antibody bamlanivimab (LY-CoV555) on hospitalization rate, all-cause mortality, and clinical outcomes of LTCF residents with pre-symptomatic, mild-to-moderate COVID-19.

## METHODS

### Setting and Population

This study included residents from three long-term care facilities (LTCFs) within the same United States health network, LECOM Health, in Erie, Pennsylvania, USA, including the LECOM Senior Living Center (SLC), LECOM at Village Square (LVS), and LECOM Health Nursing and Rehabilitation Center (LNR). Medical records of residents identified as SARS-CoV-2 positive between 1 November 1, 2020, and December 30, 2020, were included in this study.

### Measures

COVID-19 was diagnosed by the presence of SARS-CoV-2 RNA in respiratory swabs. RNA was detected via the Abbott ID Now SARS-CoV-2 reverse-transcription polymerase chain reaction (PCR) high-throughput assay or the Cepheid Xpert Xpress SARS-CoV-2 assay, conducted at the LECOM Health Senior Living Center Laboratory, supported by the Regional Response Health Collaborative (RRHC) Program of the Pennsylvania Department of Human Services, Pennsylvania Department of Health, Pennsylvania Emergency Management Agency, and additional health systems RRHC Program, 2020).

### Data Collection

Data was extracted from the electronic medical record BlueStep Platform (BlueStep, Farmington, UT, USA), and a spreadsheet was electronically created using a licensed version of Microsoft Excel, V.2016 (Microsoft Corporation, Redmond, Washington, USA). Data collected included demographic information (age, gender, race, ethnicity), date of SARS-CoV-2 positive test, emergency department (ED) visit(s), hospital admission(s), adverse effect(s), and mortality. Data collection from medical records of LTCF residents who tested positive for COVID-19 occurred between December 2020 and March 2021. No study participant randomization was conducted due to a retrospective study design.

### Definitions

Asymptomatic and pre-symptomatic disease were defined as the presence of SARS-CoV-2 RNA in respiratory swabs with absence of symptoms. Early illness was defined as the presence of two or more of the following features: fever, chills, rigors, myalgia, headache, diarrhea, sore throat, rhinorrhea. Mild-to-moderate COVID-19 was defined as illness with temperature of >100.4º F (>38º C) and/or one or more clinical findings of lower respiratory illness (e.g., cough, shortness of breath, difficulty breathing).

### Inclusion and Exclusion Criteria

In this retrospective analysis, all study participants were LTCF residents who tested positive for SARS-CoV-2. Participants were included if asymptomatic or with mild to moderate symptoms for COVID-19. The investigators reviewed the symptoms of disease and inclusion and exclusion criteria.

Inclusion criteria:

1. Mild to moderate COVID-19 resident of a nursing home with positive results of direct SARS-CoV-2 viral testing, and who are at high risk for progressing to severe COVID-19 and/or hospitalization and death.
2. High risk is defined as patients who meet at least one of the following criteria:
  1. Have a body mass index (BMI) ≥35
  2. Have chronic kidney disease
  3. Have diabetes
  4. Have immunosuppressive disease
  5. Are currently receiving immunosuppressive treatment
  6. Are ≥65 years of age
  7. Are ≥55 years of age AND have o cardiovascular disease, OR hypertension, OR chronic obstructive pulmonary disease/other chronic respiratory disease.

Exclusion criteria:

1. Patients hospitalized due to COVID-19, OR who require oxygen therapy due to COVID-19, OR who require an increase in baseline oxygen flow rate due to COVID-19 in those on chronic oxygen therapy due to underlying non-COVID-19 related comorbidity.
2. Anyone who does not meet inclusion criteria

Each patient who qualified and provided consent received a single intravenous infusion of LY-CoV555 over approximately 1 hour at a dose of 700 mg early in the disease at the long-term care facility. No additional inclusion criteria were created.

### Outcomes

Major clinical outcomes were defined as COVID-19 related in-patient hospitalization, a visit to the emergency department, and death. Since most emergency department visits resulted in hospital admissions, the authors refer to a composite of emergency department visits and in-patient hospitalizations as hospitalizations. Key secondary outcomes include safety assessments and adverse events.

### Statistical Analyses

Microsoft Excel V.2016 (Microsoft Corporation, Redmond, Washington, USA) was used to calculate relative risk of hospitalization or death among residents who received or did not receive bamlanivimab organized in 2 x 2 contingency tables. Statistical significance of comparisons between relative risk values was calculated using Fisher’s exact test in Excel. Statistical significance was set at P<0.05. Descriptive statistics in Excel was used to analyze the demographic data.

### Ethical considerations

This study was determined by the Lake Erie College of Osteopathic Medicine (LECOM) Health Institutional Review Board (IRB) to be secondary research using identifiable private information, regulated under the Health Insurance Portability and Accountability Act (HIPAA) and, therefore, was exempt from the requirement for full IRB review. No financial compensation or incentive was provided to residents.

## RESULTS

### Study participants

A total of 107 LTCF residents diagnosed with mild-to-moderate COVID-19 were evaluated from November 1, 2020, and December 31, 2020 at the LECOM Senior Living Center (SLC), LECOM at Village Square (LVS), and LECOM Health Nursing and Rehabilitation Center (LNR). The mean age of the residents was 76.8 (±13) years, 46 (43.0%) were male, 61 (57%) were female. With regard to race, 91 (85.0%) residents identified as white, 15 (14.3%) identified as Black or African American, and 1 (0.9%) identified as Other. Detailed demographic data and clinical outcomes of all study participants are displayed in Table 1.

**Table 1:** Demographic characteristics of all participants compared with hospitalization and mortality.

ALL PARTICIPANTS						
Characteristic	All Participants		Hospitalizations		Deaths	
	n = 107		n = 34		n = 23	
**Sex**	n	%	n	%	n	%
**Male**	46	42.99	18	52.94	13	56.52
**Female**	61	57.01	16	47.06	10	43.48
**Age**						
**Mean**	76.83		80.74		80.26	
**Median**	76.00		79.50		79.00	
**< 65**	21	19.63	3	8.82	2	8.70
**65 - 74**	29	27.10	8	23.53	7	30.43
**75 - 84**	24	22.43	9	26.47	6	26.09
**85 - 94**	22	20.56	8	23.53	5	21.74
**≥ 95**	11	10.28	6	17.65	3	13.04
**Ethnicity**						
**White**	91	85.05	29	85.29	20	86.96
**African American**	15	14.02	5	14.71	3	13.04
**Other**	1	0.93	0	0	0	0

Of the 107 LTCF residents diagnosed with mild-to-moderate COVID-19, 61 were offered a single intravenous infusion of neutralizing antibody LY-CoV555 (bamlanivimab) treatment in one dose of 700 mg (Table 2). Of these, 44 (72.1%) residents consented to treatment, of whom 39 (88.6%) received the full dose and 5 (11.4%) received an incomplete dose because the infusion was interrupted from intravenous line complications (Table 2). Although 61 COVID-19 positive LTCF residents were offered bamlamivimab treatment, refusal to consent occurred among 17 (27.9%) of the LTCF residents (Table 2). Of the 107 LTCF residents diagnosed with COVID-19, 46 (42.9%) residents were included in this study to whom the treatment was not offered due to unavailability of treatment (Table 3). Therefore, a total number of 63 study participants did not receive bamlamivimab treatment and served as the statistical control. These 63 participants either refused treatment (17) (Table 2) or were not offered treatment (46) (Table 3). Table 4 summarizes the clinical outcomes of all study participants.

**Table 2A:** Demographic characteristics and patient outcomes of participants offered treatment with bamlanivimab.

PARTICIPANTS OFFERED TREATMENT						
Characteristic	Offered Treatment		Hospitalizations		Deaths	
	n = 61		n = 13		n = 9	
**Sex**	n	%	n	%	n	%
**Male**	21	34.43	4	30.77	3	33.33
**Female**	40	65.57	9	69.23	6	66.67
**Age**						
**Mean**	77.45		84.00		81.22	
**Median**	76.00		86.00		80.00	
**< 65**	10	16.39	1	7.69	0	0
**65 - 74**	19	31.15	2	15.38	4	44.44
**75 - 84**	13	21.31	3	23.08	2	22.22
**85 - 94**	14	22.95	4	30.77	2	22.22
**≥ 95**	5	8.20	3	23.08	1	11.11
**Ethnicity**						
**White**	52	85.25	11	84.62	9	100
**African American**	9	14.75	2	15.38	0	0
**Other**	0	0	0	0	0	0

**Table 2B:** Demographic characteristics and patient outcomes of participants offered treatment with bamlanivimab compared with consent, treatment dosage, hospitalization, and mortality (H* = Hospitalizations).

PARTICIPANTS OFFERED TREATMENT																		
Characteristic	Consent + Full Treatment		H*		Deaths		Consent + Incomplete Treatment		H*		Deaths		Did Not Consent ? No Treatment		H*		Deaths	
	n = 39		n = 5		n = 4		n = 5		n = 3		n = 1		n = 17		n = 5		n = 4	
**Sex**	n	%	n	%	n	%	n	%	n	%	n	%	n	%	n	%	n	%
**Male**	11	28.21	0	0	1	25.00	2	40.00	1	33.33	1	100	8	47.06	3	60.00	1	25.00
**Female**	28	71.79	5	100	3	75.00	3	60.00	2	66.67	0	0	9	52.94	2	40.00	3	75.00
**Age**																		
**Mean**	76.00		86.80		83.25		86.60		94.67		93.00		74.35		74.80		76.25	
**Median**	76.00		86.00		80.00		92.00		93.00		93.00		72.00		80.00		76.50	
**< 65**	7	17.95	0	0	0	0	1	20.00	0	0	0	0	2	11.76	1	20.00	0	0.00
**65 - 74**	11	28.21	1	20.00	2	50.00	0	0	0	0	0	0	8	47.06	1	20.00	2	50.00
**75 - 84**	10	25.64	1	20.00	0	0	0	0	0	0	0	0	3	17.65	2	40.00	2	50.00
**85 - 94**	7	17.95	1	20.00	1	25.00	3	60.00	2	66.67	1	100	4	23.53	1	20.00	0	0.00
**≥ 95**	4	10.26	2	40.00	1	25.00	1	20.00	1	33.33	0	0	0	0.00	0	0	0	0
**Ethnicity**																		
**White**	35	89.74	5	100	4	100	5	100	3	100	1	100	12	70.59	3	60.00	4	100
**African American**	4	10.26	0	0	0	0	0	0	0	0	0	0	5	29.41	2	40.00	0	0
**Other**	0	0	0	0	0	0	0	0	0	0	0	0	0	0	0	0	0	0

**Table 3:** Demographic characteristics and patient outcomes of participants not offered treatment with bamlanivimab compared with hospitalization and mortality.

PARTICIPANTS NOT OFFERED TREATMENT						
Characteristic	Not Offered Treatment		Hospitalizations		Deaths	
	n = 46		n = 21		n = 14	
**Sex**	n	%	n	%	n	%
**Male**	25	54.35	14	66.67	10	71.43
**Female**	21	45.65	7	33.33	4	28.57
**Age**						
**Mean**	76.44		78.71		79.64	
**Median**	74.00		77.00		78.00	
**< 65**	11	23.91	2	9.52	2	14.29
**65 - 74**	10	21.74	6	28.57	3	21.43
**75 - 84**	11	23.91	6	28.57	4	28.57
**85 - 94**	8	17.39	4	19.05	3	21.43
**≥ 95**	6	13.04	3	14.29	2	14.29
**Ethnicity**						
**White**	39	84.78	18	85.71	11	78.57
**African American**	6	13.04	3	14.29	3	21.43
**Other**	1	2.17	0	0	0	0

### Primary Outcomes

#### Hospitalization

**Figure 1.**
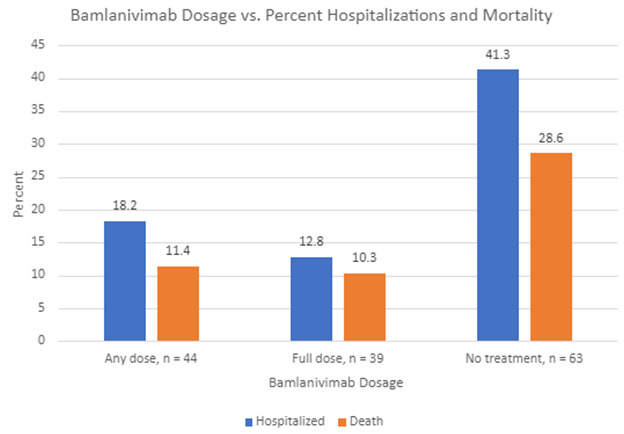
Comparison of bamlanivimab dosage (full dose 700 mg, any dose - full or partial dose, no treatment) with percent hospitalization and mortality.

Concerning hospitalization rate, among the 107 LTCF residents diagnosed with COVID-19, residents who received a 700 mg dose of bamlanivimab experienced a lower relative risk for hospitalization, including visits to the emergency room, compared to residents who did not receive bamlanivimab treatment (RR = 0.31, P = 0.0035). Of the 39 LTCF residents who received a full dose, only 5 (13%) were subsequently admitted to the hospital. Conversely, for the 63 LTCF residents who did not receive bamlanivimab, 26 (41.3%) were subsequently hospitalized (Table 4). Five additional residents received an incomplete dose because the infusion was interrupted from intravenous line complications. These five residents, nonetheless, received a partial dose. Collectively, of the 44 LTCF residents who received either a full or partial dose of bamlanivimab, 8 (18%) were admitted to the hospital compared to 41.3% hospitalization rate for residents (26 out of 63) who did not receive bamlanivimab treatment (RR=0.44, P=0.0124) (Table 4 and Fig. 1).

Concerning age, of the 34 LTCF residents who were hospitalized and received either the full treatment or no treatment, 3 were under the age of 65 years, while 31 were 65 years of age or older. For the 21 LTCF residents under the age of 65, 7 received full treatment, none (n=0) of whom were hospitalized, and 13 received no treatment, 3 of whom were hospitalized (Table 5). The relative risk of hospitalization for LTCF residents with mild to moderate COVID-19, however, was not statistically significant between full treatment and no treatment groups for residents under the age of 65 (RR=0.40, P=0.0686).

Concerning gender, of the 46 male LTCF residents who received either the full treatment or no treatment, 18 were hospitalized. Of the 61 female LTCF residents who received either the full treatment or no treatment, 16 were hospitalized. The relative risk of hospitalization for LTCF residents with mild to moderate COVID-19, however, was not statistically significant between full treatment and no treatment groups for residents based on gender (RR=1.49, P=0.2084).

**Table 4:** COVID-19 positive participants compared with bamlanivimab dosage, hospitalization and mortality.

	Total	Hospitalizations		Deaths	
	n	n	%	n	%
**COVID-19 positive patients**	107	34	31.78	23	21.50
**Offered treatment**	61	13	21.31	9	14.75
**Consent to treatment**	44	8	18.18	5	11.36
**Full dose**	39	5	12.82	4	10.26
**Incomplete dose**	5	3	60.00	1	20.00
**Refused treatment**	17	5	29.41	4	23.53
**Not offered treatment**	46	21	45.65	14	30.43

#### Mortality

Concerning mortality among LTCF residents diagnosed with COVID-19, those residents treated with a 700 mg dose of bamlanivimab also demonstrated a lower relative risk of death compared to those residents who were not treated with bamlanivimab (RR= 0.36, P=0.0458). Of the 39 residents who received a full dose, 4 (10%) died. Conversely, of the 63 residents who did not receive bamlanivimab, 18 (29%) died. As previously described, five additional patients received a partial dose. Collectively, of the 44 patients who received either a full or partial dose, 5 (11%) died compared with 18 out of 63 who died and did not receive bamlanivimab treatment (no full or partial dose) (RR=0.39, P=0.0541) (Fig. 1).

Concerning age, of the 23 LTCF residents who died and received either the full treatment or no treatment, 2 were under the age of 65 years, while 21 were 65 years of age or older. For the 2 LTCF residents under the age of 65, both (n=2) received no treatment and subsequently died (Table 5). The relative risk of death for LTCF residents with mild to moderate COVID-19, however, was not statistically significant between full treatment and no treatment groups for residents under the age of 65 (RR=0.39, P=0.2339).

Concerning gender, of the 46 male LTCF residents who received either the full treatment or no treatment, 13 died. Of the 61 female LTCF residents who received either the full treatment or no treatment, 10 died. The relative risk of death for LTCF residents with mild to moderate COVID-19, however, was not statistically significant between full treatment and no treatment groups for residents based on gender (RR=1.72, P=0.1594).

Therefore, administration of bamlanivimab to LTCF residents who tested positive for COVID-19 positively impacted hospitalization and death, but its greatest impact was on reducing hospitalization, but lost statistical significance for reducing mortality if a partial dose was utilized. Additionally, when hospitalization and mortality were compared among age and gender treatment subgroups, no statistically significant differences were observed.

**Table 5:** **Hospitalization and mortality by age and gender of participants who received full treatment with bamlanivimab compared with participants not treated with bamlanivimab**. Participants who received a partial dose are not included in this table.

HOSPITALIZATION AND MORTALITY BY AGE AND GENDER						
Characteristic	All Participants		Hospitalizations		Deaths	
	n = 107		n = 34		n = 23	
**Sex**	n	%	n	%	n	%
**Male**	46	42.99	18	52.94	13	56.52
**Full treatment**	11	23.91	0	0	1	7.69
**No treatment**	33	71.74	17	94.44	11	84.62
**Female**	61	57.01	16	47.06	10	43.48
**Full treatment**	28	45.90	5	31.25	3	30.00
**No treatment**	30	49.18	9	56.25	7	70.00
**Age**						
**< 65**	21	19.63	3	8.82	2	8.70
**Full treatment**	7	33.33	0	0	0	0
**No treatment**	13	61.90	3	100.00	2	100.00
**65 or older**	86	80.37	31	82.35	21	91.3
**Full treatment**	32	37.21	5	16.13	4	19.05
**No treatment**	50	58.14	23	74.19	16	76.19

### Secondary Outcome

Of the 44 LTCF residents who tested positive for COVID-19 and received bamlanivimab infusion treatment, 5 residents (11.36%) experienced adverse effects. The mean age of residents experiencing adverse effects was 76.6 years and all were female. Of the 5 LTCF residents who experienced adverse effects, four (4) had received the full dose, and one (1) had received an incomplete dose. Three (3) residents reported only one adverse effect while two (2) residents reported two adverse effects. The most common adverse effect was nausea, which was reported by 4 of the 5 (80%) residents. Other adverse effects reported by three different residents included vomiting, diarrhea, and dizziness, each only reported once. Only one of the residents who reported an adverse effect was hospitalized. None of the residents who reported an adverse effect died (Table 6).

**Table 6:** Adverse effects following bamlanivimab infusion.

Participant Consent and Dosage		Adverse Effects, n (%)	Nausea	Vomiting	Diarrhea	Dizziness	Hospitalizations	Death	Age, Mean
**Consented and received treatment**	44	5 (11.36%)	4	1	1	1	1	0	76.6
**Full dose**	39	4 (10.26%)	3	1	1	1	0	0	71
**Incompletedose**	5	1 (20.00%)	1	0	0	0	1	0	99

## DISCUSSION

In this retrospective study, investigators examined the efficacy of bamlanivimab (LY-CoV555) in the treatment of pre-symptomatic, mild-to-moderate COVID-19 in long term care facility residents. The aim of this study was to evaluate the effectiveness of early intervention neutralizing monoclonal antibody therapy on hospitalization, death, and adverse side effects. Data analysis revealed greater benefit to LTCF residents with the use of bamlanivimab across primary and secondary clinical outcomes.

LTCF residents with or without underlying medical conditions are at increased risk for severe COVID-19 disease (CDC, 2020). Among LTCF residents who received treatment with the complete 700 mg dose infusion of bamlanivimab, the percentages of hospitalization and death were significantly lower than in those who did not receive the infusion of bamlanivimab. Since severity of illness is primarily driven by lung injury from SARS-CoV-2 infection in the lower respiratory tract, the effect of bamlanivimab (LY-CoV555) therapy on clinical outcomes, specifically the frequency of hospitalization, is an important outcome given the association between hospitalization and subsequent mortality in patients with COVID-19 [3,4]. In addition, no serious adverse events were recorded. These results provide support for the potential of neutralizing monoclonal antibody therapy to reduce both the risk of progression to severe disease and the severity of disease among (LTCF) residents with symptomatic COVID-19 with an acceptable safety profile.

Several studies have shown that neutralizing antibodies have an important role in the protection or recovery from many viral infections. This study supports previous data of the clinical benefit of monoclonal antibodies in COVID-19. Despite the huge increase in the number of management articles published during the pandemic, we consider our study tabulate research in an extremely high-risk population residing in a nursing home with ongoing COVUD-19 outbreak in a time where there was not yet available treatment, vaccines or prevention. We managed to get the infusions in the midst of a second wave of the pandemic and used promptly in our (LTCF) residents with a positive result.

Unfortunately, the sustained increase of SARS-CoV-2 viral variants that are resistant to bamlanivimab alone has resulted in the increased risk for treatment failure. The FDA has determined that the known and potential benefits of bamlanivimab, when administered alone, no longer outweigh the known and potential risks for its authorized use. Therefore, the agency determined that the criteria for issuance of an emergency use authorization were no longer met, and on April 16, 2021, revoked its emergency use authorization for the treatment of mild-to-moderate COVID-19. (FDA Revokes Emergency Use Authorization for Monoclonal Antibody Bamlanivimab, 2021). This medication alone is currently not in use. The FDA emergency use authorization for the combination of bamlanivimab with etesevimab, as well as other monoclonal antibodies alone (sotrovimab) or in combination (casirivimab/imdevimab).

This study has several limitations. The emergence of viral variants was not accounted for in the analyses. Additionally, no immunosuppressed residents were included in the study. While the date of hospital admission was objectively verifiable, the date of symptom onset was self-reported and may be subject to study participant recall bias. Also, the study group of 5 patients who received a partial dose of bamlanivimab treatment is relatively small. Although only 16 of 107 residents (15%) were identified as non-white and is relatively small, this proportion is representative of the general population of Erie, Pennsylvania, USA. Cause of death and severity of illness were unable to be determined based on available documentation in the electronic medical record.

## CONCLUSION

The association between intravenous administration of monoclonal antibody bamlanivimab (LY-CoV555) to LTCF residents recently diagnosed with pre-symptomatic, mild-to-moderate COVID-19 and are considered high risk for disease progression was statistically significant with hospitalization and mortality. COVID-19 positive LTCF residents who received an intravenous administration of a full dose of bamlanivimab antibody treatment were associated with a statistically significant lower relative risk for hospitalization and death compared to those LTCF residents who did not receive a full dose. For those LTCF residents who received a partial dose bamlanivimab antibody treatment, the relative risk for hospitalization remained statistically lower than those residents who did not receive any treatment, while the relative risk for mortality became not statistically associated. Therefore, a full dose of bamlanivimab antibody treatment is recommended to reduce the relative risk of hospitalization and mortality among LTCF COVID-19 patients. Neutralizing monoclonal antibodies directed at the receptor-binding domain of SARS-CoV-2 spike protein are a potential therapeutic option for the COVID-19 positive, mild-to-moderate disease, LTCF population. Monoclonal antibody treatment provides immediate, passive immunity and may limit disease progression and complications in these patients. The monoclonal antibody was well-tolerated.
